# Interspecific and Intraspecific Transcriptomic Variations Unveil the Potential High-Altitude Adaptation Mechanisms of the *Parnassius* Butterfly Species

**DOI:** 10.3390/genes15081013

**Published:** 2024-08-01

**Authors:** Chen Ding, Chengyong Su, Yali Li, Youjie Zhao, Yunliang Wang, Ying Wang, Ruie Nie, Bo He, Junye Ma, Jiasheng Hao

**Affiliations:** 1 College of Life Sciences, Anhui Normal University, Wuhu 241002, China; 15956625813@163.com (C.D.); sky475342@163.com (C.S.); 18214812293@163.com (Y.L.); bioala@ahnu.edu.cn (Y.Z.); wyl1119@163.com (Y.W.); wy5991139@ahnu.edu.cn (Y.W.); niere2021@ahnu.edu.cn (R.N.); hebo90@126.com (B.H.); 2 College of Physical Education, Anhui Normal University, Wuhu 241002, China; 3 Nanjing Institute of Geology and Paleontology, Chinese Academy of Sciences, Nanjing 210008, China; jyma@nigpas.ac.cn

**Keywords:** *Parnassius* butterflies, transcriptome, high-throughput sequencing, alpine environment, adaptative evolution

## Abstract

*Parnassius* butterflies have significantly advanced our understanding of biogeography, insect–plant interactions, and other fields of ecology and evolutionary biology. However, to date, little is known about the gene expression patterns related to the high-altitude adaptation of *Parnassius* species. In this study, we obtained high-throughput RNA-seq data of 48 adult *Parnassius* individuals covering 10 species from 12 localities in China, and deciphered their interspecific and intraspecific expression patterns based on comparative transcriptomic analyses. Though divergent transcriptional patterns among species and populations at different altitudes were found, a series of pathways related to genetic information processing (i.e., recombination, repair, transcription, RNA processing, and ribosome biogenesis), energy metabolism (i.e., oxidative phosphorylation, thermogenesis, and the citrate cycle), and cellular homeostasis were commonly enriched, reflecting similar strategies to cope with the high-altitude environments by activating energy metabolism, enhancing immune defense, and concurrently inhibiting cell growth and development. These findings deepen our understanding about the molecular mechanisms of adaptative evolution to extreme environments, and provide us with some theoretical criteria for the biodiversity conservation of alpine insects.

## 1. Introduction

The mechanisms of evolution and related environmental adaptation are core issues in the field of evolutionary biology. Gene expression patterns revealed by transcriptional changes are important molecular phenotypes of key genes closely related to environmental adaptation. Increasing evidence has shown that the high adaptability of insects to counter harsh environmental challenges is closely related to the complex functional pathways and regulatory networks of a series of genes or gene families through gene transcription regulation [1,2,3,4,5]. For example, significantly enhanced or suppressed pathways mainly related to energy production, mitochondrial biogenesis, clearance of dysfunctional proteins, and metabolic processes have been identified in the migratory locust (*Locusta migratoria*) and the ghost moth (*Thitarodes pui*) when exposed to short-term hypoxia or normoxia, respectively [6,7,8]. Diapause-linked gene expression patterns with a series of pathways and duplicated genes mainly responsible for hormone biosynthesis, energy metabolism, and immune defense are likely to correlate with local adaptation in adult *Parnassius glacialis* populations [1]. Moreover, expression levels for nine mitochondrial genes in nine different developmental stages were also significantly suppressed in the grassland moth *Gynaephora alpherakii* at a high elevation (~4800 m above sea level) [9]. In general, current research on the mechanisms of environmental adaptation in insect groups primarily examines individual species subjected to short-term artificial stressors, such as hypoxia or normoxia and low or high temperatures, neglecting the synergistic impacts of multiple environmental factors in natural settings; however, gene expression influencing factors are more difficult to identify for insects living in fluctuating natural environments than for those in controlled laboratory settings [10].

Butterflies are particularly sensitive to various environmental factors, such as climatic changes, and some of them have become the ideal organisms for fundamental studies in biogeography, insect–plant interactions, and other fields of ecology and evolutionary biology [11,12,13,14,15]. The genus *Parnassius* (Papilionidae: Parnassiinae: Parnassiini) is particularly noteworthy for its relatively rich species diversity and alpine distribution pattern [16]. The majority of species in this genus often occur in sympatry, and the same species can colonize at different localities across a broad elevational range of 2800–5200 m above sea level on the Qinghai-Tibet Plateau (QTP) and adjacent mountainous regions, feeding mostly on the Crassulaceae and Papaveraceae plants at the larval stage [1,17,18]. Prior research indicated that the *Parnassius* began to originate in the Middle Miocene, being adapted to harsh environmental conditions, such as cold temperatures, low oxygen levels, and intense ultraviolet radiation. Their later diversification has been linked to host plant shifts, ancient gene introgression, and rapid genomic expansion [11,19,20,21,22,23]. However, most of the previous studies on this genus mainly involved molecular phylogeny, phylochronology, and historical biogeography, while those on the gene expression patterns related to high-altitude adaptation remain unclear, and, in particular, transcriptomic analysis based on reference genomes to explore their interspecific and intraspecific genome-wide expression patterns has not been reported.

In this study, the transcriptomes of a total of 48 adult individuals from 10 representative *Parnassius* species (*Parnassius nomion*, *P*. *epaphus*, *P*. *mercurius*, *P*. *cephalus*, *P*. *imperator*, *P*. *andreji*, *P*. *simo*, *P*. *orleans*, *P*. *stubbendorfii*, and *P. glacialis*) were newly determined through high-throughput sequencing technology. Using the chromosome-level genomic data of *P. glacialis* and *P. cephalus* as the references, we calculated transcriptomic expression matrices for each sample and carried out large-scale comparative analyses through several strategies to decipher the interspecific and intraspecific expression patterns of the different altitude-distributed *Parnassius* butterflies.

## 2. Materials and Methods

### 2.1. Sample Collection

**Figure 1:**
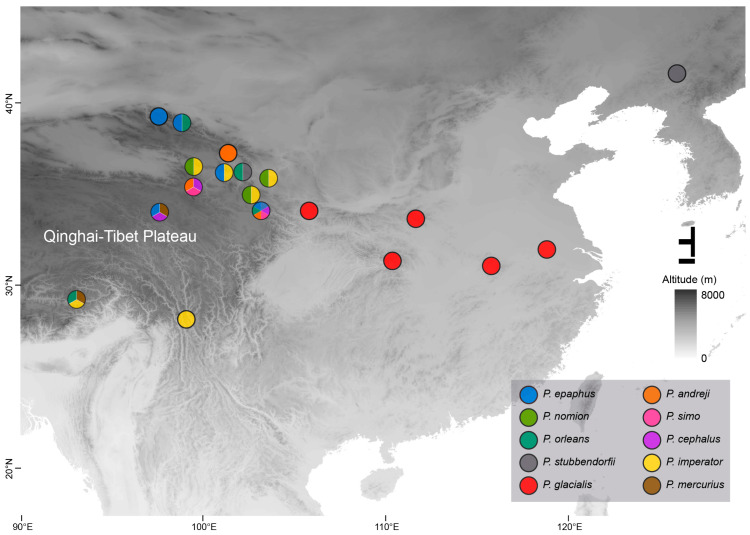
The sampling localities of the *Parnassius* samples in this study.

All imago individuals (*n* = 48) representing 10 *Parnassius* butterfly species at 12 localities were field collected between 10:00 and 13:00 in the daytime, mostly in mountain areas on the Qinghai-Tibet Plateau in China during April to July 2020 (Figure 1, Table S1). All samples were initially preserved in RNA stabilization solution (Sangon Biotech, Shanghai, China) in the field and transferred to −80 °C until RNA extraction. Muscle tissues from the thorax of three individuals per sample were used for purified RNA extraction. According to the elevations of the sampling locality (Table S1), the samples here were divided into three groups, classified as the low-altitude group (LA, including *P. glacialis* and *P. stubbendorfii*, 500–1700 m), the medium-altitude group (MA, including *P. imperator* and *P. nomion*, 2400–3700 m), and the high-altitude group (HA, all other species, including *P. epaphus*, *P. mercurius*, *P. simo*, *P. andreji*, *P. orleans,* and *P. cephalus*, 3900–4500 m).

### 2.2. mRNA-Seq Library Construction, Illumina Sequencing, and Gene Abundance Estimation

The library construction and illumina sequencing and assembly were performed following the methods in a previous study [21]. Specifically, total RNA was extracted using Trizol Reagent (Invitrogen, Carlsbad, CA, USA) according to the manufacturer’s instructions. The mRNA-containing polyA structures were enriched using Oligo (dT) magnetic beads (Shanghai Personal Biotechnology Co. Ltd., Shanghai, China). The quality and integrity of the mRNA were determined using a NanoDrop spectrophotometer (Thermo Scientific, Barrington, IL, USA) and Agilent 2100 Bioanalyzer (Agilent Technologies, Palo Alto, CA, USA). The sequencing library was paired-end sequenced with an average sequencing depth > 67 million reads per sample, based on the Illumina HiSeq2500 Sequencing platform (2 × 150 bp, Shanghai Personal Biotechnology Co. Ltd., Shanghai, China).

After the adaptor paired reads were removed using cutadapt v.1.2.1, FastQC v.0.11.9 was used for quality control of the transcriptome data to ensure data quality and reliability [24,25], and reads of less than 50 bp and ambiguous nucleotides were removed. These high-quality sequences were then mapped with the software TBtools v.2.0 [26], using *P. glacialis* [23] and *P. cephalus* (unpublished) genomes as the reference. Based on the mapping results, the expression levels of each gene were calculated.

### 2.3. Multiple Regression Analysis Based on Distance Matrix

To investigate the impact of various environmental factors on the gene expression patterns of *Parnassius*, Multiple Regression on Distance Matrices (MRM) [27] were performed with the ecodist v2.0.9 R package [28] to assess the relative contributions of altitude, geographic location, and host plants on the gene expression patterns. The Jaccard and Bray–Curtis distance matrices for MRM analysis were used to enhance the credibility of the results [29]. The gene expression levels of the genus *Parnassius* were used as the dependent variable, while the altitude, geographic location, and host plant were used as three independent variables. Venn diagrams were used to illustrate the relative effects of the independent variables on the dependent variable.

### 2.4. Differential Gene Expression Analysis between Parnassius Species or Intraspecific Populations

Differential gene expression analysis was conducted to identify the similarities and differences in gene expression patterns among *Parnassius* species. We divided 10 representative *Parnassius* species according to altitude into a low-altitude group (LA, including *P. glacialis* and *P. stubbendorfii*), a medium-altitude group (MA, including *P. imperator* and *P. nomion*), and a high-altitude group (HA, including *P. epaphus*, *P. mercurius*, *P. simo*, *P. andreji*, *P. orleans*, and *P. cephalus*). The analysis focused on three differentially expressed gene datasets, which were obtained from comparisons between the LA vs. HA, LA vs. MA, and MA vs. HA groups. Genes were considered differentially expressed genes (DEGs) if they had an expression change with |log_2_FoldChange| ≥ 1 and a Benjamini–Hochberg adjusted *p*-value < 0.05, as determined by DESeq2 and edgeR based on the read count data [30,31,32]. To minimize false positives, only the DEGs identified by both DESeq2 and edgeR in each pairwise comparison were kept for further analysis. Moreover, the number of DEGs which passed |log_2_FoldChange| > 2 and had a Benjamini–Hochberg adjusted *p*-value < 0.05 was also calculated for each pairwise comparison. Gene functions from the reference genomes (*P. glacialis* and *P. cephalus*) were assigned according to the best match by aligning the protein sequences to the Swiss-Prot and the Kyoto Encyclopedia of Genes and Genome (KEGG) using Blastp (with the threshold of E-value ≤ 1 × 10^−5^), followed by the KEGG enrichment analysis of DEGs using TBtools v.2.0, with an adjusted *p*-value < 0.05 as the significance threshold [2,33,34].

In order to reveal the differentially expressed genes closely associated with high-altitude adaptation under the same genetic background, the intraspecific comparisons herein were focused on four *Parnassius* species sampled at different altitudes, namely, the And_el vs. And_my, Sim_el vs. Sim_bc, Nom_ck vs. Nom_bm, and Epa_hs vs. Epa_gg.

### 2.5. Weighted Gene Co-Expression Network Analysis (WGCNA)

A weighted gene co-expression network analysis (WGCNA) on the Majorbio Cloud platform serves as an exploratory tool to identify highly interconnected gene clusters in various modules across samples through unsupervised clustering [35]. We conducted a WGCNA to identify key gene clusters and then correlate their expression patterns to the subgenus of *Parnassius* species to further reveal the common expression pattern of different species of *Parnassius* at different altitudes involved in environmental adaptations, with the following parameters: unsigned for TOMType, 30 for minModuleSize, 0.35 for mergeCutHeight, and default values for the other parameters.

### 2.6. Validation of Gene Expression by RT-qPCR

To validate the expression patterns derived from our RNA-seq analysis, 10 genes, including both DEGs and non-DEGs among the representative samples covering a total of nine *Parnassius* species (exclusive of the *P. mercurius* for its only one individual), were selected for real-time reverse transcription (RT) quantitative PCR analysis (the primers are listed in Table S2). Reversed cDNA was synthesized using the PrimeScript™ 1st stand cDNA Synthesis Kit (Takara, Shanghai, China) from the total RNA isolated as described above. All RT-qPCR experiments were run in triplicate using the LightCycler 480 II (Roche Diagnostics, Basel, Switzerland) with SYBR green (Vazyme, Nanjing, China), based on the following cycling parameters: 95 °C for 5 min, 40 cycles of 95 °C for 15 s, and 60 °C for 30 s. The 2^−ΔΔCT^ method was used for the analysis of the relative gene expression data [36], and the elongation factor 1 α (EF1-α) gene was chosen as the reference gene [37]. The R values of Spearman’s correlation coefficient were calculated to represent the correlations between the data obtained from the RT-qPCR and RNA-seq.

## 3. Results

### 3.1. Transcriptome Sequencing Data Quality

To investigate the molecular mechanisms involved in the high-altitude adaptation of *Parnassius* species, RNA-seq analysis was conducted on a total of 48 samples from 12 localities. Each sample yielded an average data volume of approximately 68.38 million reads, resulting in a total base count of approximately 10.26 Gbp. The data revealed that, on average, 97.74% and 93.72% of bases harbored an accuracy index exceeding 99% (>Q20) and 99.9% (>Q30), respectively (Table S3).

### 3.2. Multivariate Regression Analysis of Distance Matrices

**Figure 2:**
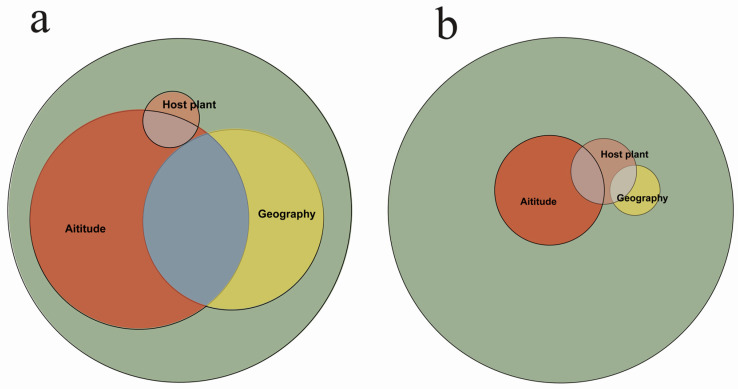
Multiple regression analysis based on altitude, geographic location, and host plant. (**a**) Modeling with Jaccard distance matrix; (**b**) modeling with Bray–Curtis distance matrix.

The distance matrix-based multivariate regression analysis showed that all three factors (altitude, geographic location, and host plant) had a statistically significant influence on gene expression patterns (*p* = 0.001). Especially, the combined effect of these factors, as measured by the Jaccard distance matrix, accounted for 25.4% of the observed gene expression differences, and among the three factors, altitude change was shown to be the most significant factor affecting the gene expressions (Figure 2, Table S4).

### 3.3. Statistics of Interspecific Differentially Expressed Genes (DEGs) and Enriched Pathways

**Figure 3:**
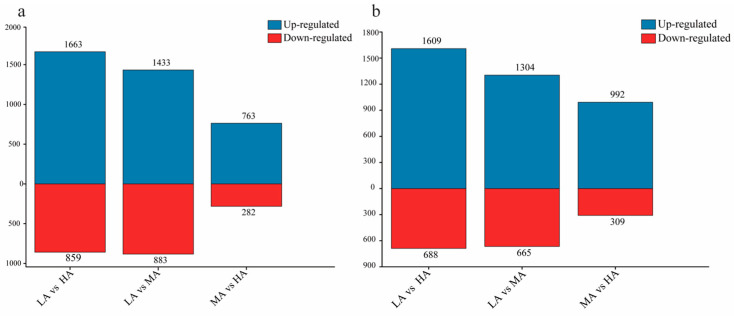
The number of differentially expressed genes for each pairwise comparison. Bar chart diagrams showing overlaps of DEGs with increased (red) or decreased (blue) transcript abundance in three pairs of comparisons. (**a**) *P. glacialis* was used as the reference genome; (**b**) *P. cephalus* was used as the reference genome.

Based on the results of each pairwise comparison, the numbers of DEGs between LA and HA (about 2500 genes) were relatively larger than the others, while those between MA and HA (about 1100–1300 genes) were shown to be smallest, regardless of whether *P. glacialis* or *P. cephalus* was used as the reference genome (Figure 3). Though the number of DEGs (about 100–400 genes) which passed |log_2_FoldChange| > 2 decreased, the trend of those for each pairwise comparison was generally consistent (Table S8). Therefore, it was speculated that the gene expression differences among *Parnassius* species were closely correlated with altitude variations.

**Figure 4:**
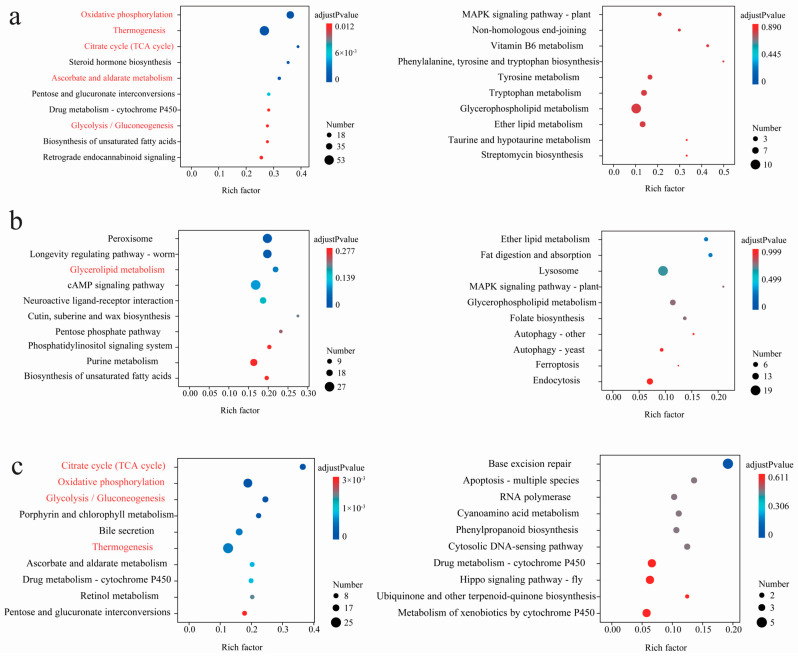
(**a**–**c**) KEGG enrichment results of the DEGs (*P. glacialis* was used as the reference genome: left, up-regulated; right, down-regulated) in pairwise comparisons of HA vs. LA, MA vs. LA, and HA vs. MA (the latter group used as the control, similarly hereinafter), respectively. For each comparison, only the top 10 pathways with the most significant enrichment are shown. The shared pathways that appear in the same pairwise comparison are highlighted in red, regardless of the reference genome (similarly hereinafter).

**Figure 5:**
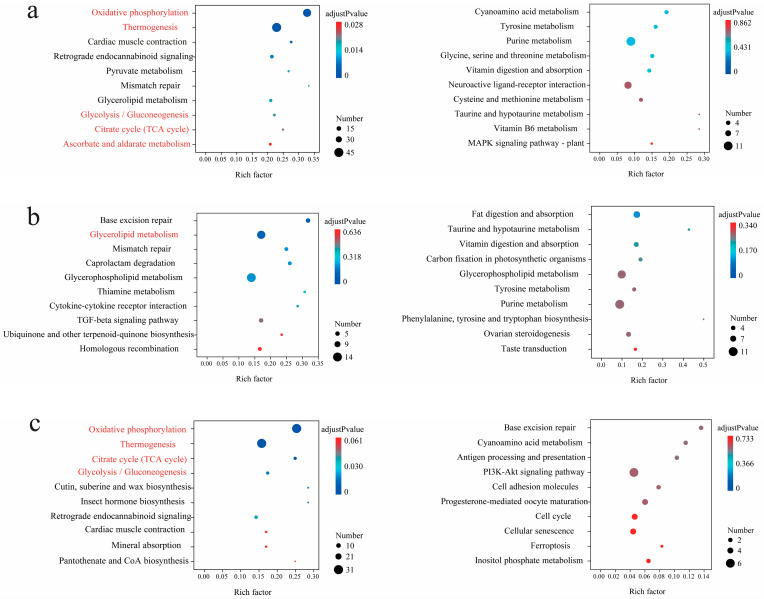
(**a**–**c**) KEGG enrichment results of the DEGs (*P. cephalus* was used as the reference genome: left, up-regulated; right, down-regulated) in pairwise comparisons of HA vs. LA, MA vs. LA, and HA vs. MA, respectively. For each comparison, only the top 10 pathways with the most significant enrichment are shown.

Interspecific comparative analysis among the *Parnassius* species generally revealed that, compared to those of the low- and moderate-altitude groups, the up-regulated DEGs for the high-altitude *Parnassius* species were primarily involved in the pathways of energy metabolism (i.e., oxidative phosphorylation, glycolysis/gluconeogenesis, and the citric acid cycle) and organic systems (i.e., heat generation, myocardial contraction, and retrograde endocannabinoid signaling), which could facilitate the energy needs and maintain body temperature by enhancing the energy supply. In addition, the insect hormone biosynthesis pathway was also shown to be activated in the high-altitude groups, and in this case was likely to facilitate *Parnassius* reproduction at high altitudes. However, for the comparison of LA vs. MA, no KEGG pathways were significantly enriched (adjusted *p*-values < 0.05, Figure 4 and Figure 5, Table S5).

### 3.4. Statistics of Intraspecific Differentially Expressed Genes (DEGs) and Enriched Pathways

**Figure 6:**
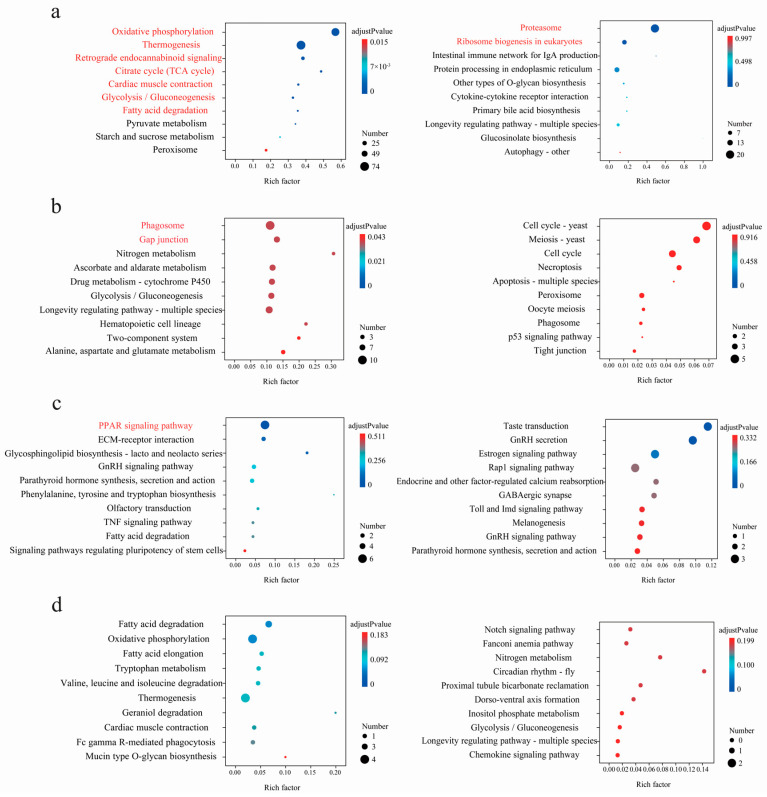
KEGG enrichment results of the DEGs (*P. glacialis* is used as the reference genome: left, up-regulated; right, down-regulated) in pairwise comparisons of And_el vs. And_my (**a**), Sim_el vs. Sim_bc (**b**), Nom_ck vs. Nom_bm (**c**), and Epa_hs vs. Epa_gg (**d**), respectively. For each comparison, only the top 10 pathways with the most significant enrichment are shown.

**Figure 7:**
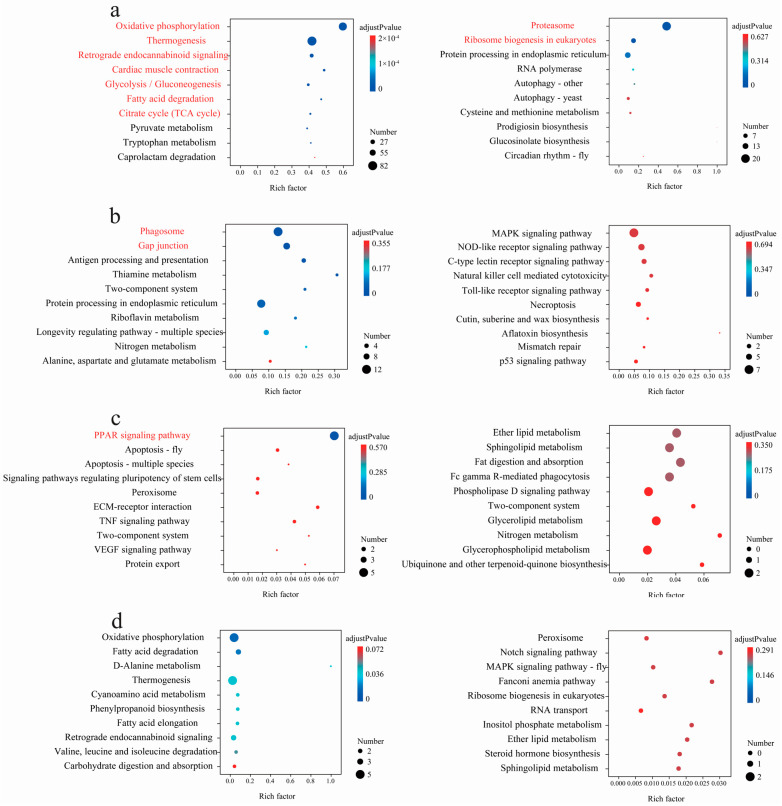
KEGG enrichment results of the DEGs (*P. cephalus* is used as the reference genome: left, up-regulated; right, down-regulated) in pairwise comparisons of And_el vs. And_my (**a**), Sim_el vs. Sim_bc (**b**), Nom_ck vs. Nom_bm (**c**), and Epa_hs vs. Epa_gg (**d**), respectively. For each comparison, only the top 10 pathways with the most significant enrichment are shown.

Based on the results of the intraspecific comparisons of DEGs, it was found that the DEGs were generally species-specific, regardless of whether *P. glacialis* or *P. cephalus* was used as the reference genome (Figure S1, Table S9). Intraspecific comparative analysis revealed that for populations at higher altitudes, up-regulated expression genes were mainly involved in metabolism, including the citric acid cycle, glycolysis, gluconeogenesis, oxidative phosphorylation, fatty acid metabolism, and starch and sucrose metabolism. Additionally, most genes related to thermogenesis and retrograde endocannabinoid signaling also presented increased expression levels. These gene expression variations might contribute to enhanced hypoxia and cold adaptation in high-altitude environments. Simultaneously, for the populations at higher altitudes, the pathways related to phagocytosis and gap junctions were significantly enriched and primarily associated with immune defense and signal transduction. Furthermore, for the populations at higher altitudes, the genes involved in the activation of the peroxisome proliferator-activated receptor signaling pathway were shown to be up-regulated, likely leading to the enhanced glucose uptake [38,39,40], while those associated with genetic information processing, including proteasome and ribosome biogenesis, were shown to be down-regulated. Generally, the results herein indicated that, in contrast to the populations at lower altitudes, the higher-altitude populations had evolved molecular adaptative strategies through gene expression regulation to cope with the extremely high elevation environments by activating energy metabolism, enhancing immune defense, and concurrently inhibiting cell growth and development (adjusted *p*-values < 0.05, Figure 6 and Figure 7 and Table S6).

### 3.5. Featured Modules Based on WGCNA

**Figure 8:**
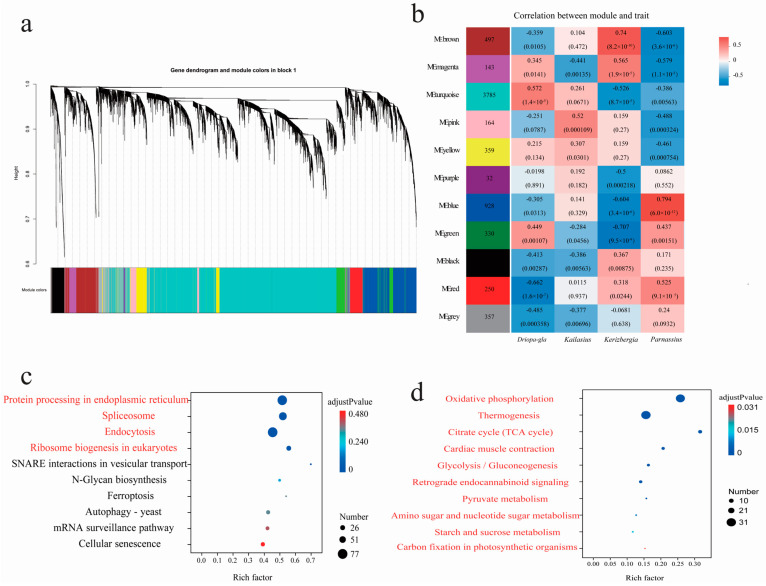
(**a**) Hierarchical clustering tree (gene dendrogram) showing 11 modules of genes co-expressed by WGCNA using *P. glacialis* as the reference genome. The major tree branches constitute 11 modules, labeled with different colors. (**b**) Module–locality relationship (each row represents a module, each column represents a specific sampling locality, and the correlation coefficient between module and locality is represented by the value in each cell at the row–column intersection, with the *p*-value shown in parentheses. (**c**,**d**) KEGG enrichment analyses of the genes in the turquoise and red modules, respectively. For each module, only the top 10 pathways with the most significant enrichment are shown (Table S7).

To further elucidate the adaptive mechanisms of the genus *Parnassius* from different subgenera to different altitudinal environments, we employed a WGCNA to identify key gene clusters and conducted KEGG functional enrichment analysis. Our results, using *P. glacialis* as the reference genome, revealed that these expressed genes were clustered into 11 major modules (Figure 8). Among them, two modules (turquoise and red) contained large number of genes and were significantly associated with several subgenera with high Spearman correlation coefficients, both of which were also moderately correlated with the subgenus *Kailasius* at median altitudes. The turquoise module, comprising 3785 genes, was significantly positively correlated with *P. glacialis* (Subgenus *Driopa*) at low altitudes (Spearman correlation coefficient = 0.572, adjusted *p*-value = 11.43 × 10^−5^), and negatively correlated with the subgenera *Kerizberga* and *Parnassius* at relatively high altitudes (Spearman correlation coefficient ≤ −0.386, adjusted *p*-value ≤ 5.63 × 10^−3^). In contrast, the red module, containing 250 genes, was positively correlated with the subgenera *Kerizberga* and *Parnassius* at relatively high altitudes (Spearman correlation coefficient ≥ 0.318, adjusted *p*-value ≤ 2.44 × 10^−2^), while negatively correlated with *P. glacialis* at low altitudes (Spearman correlation coefficient = −0.662, adjusted *p*-value = 1.65 × 10^−7^) (Figure 8). The KEGG enrichment analysis revealed that the genes in the turquoise module mainly participated in biological processes, such as protein processing in the endoplasmic reticulum, ribosome biogenesis, and spliceosome and endocytosis, which are closely related to cell growth and developmental regulation, while the genes in the red module were primarily involved in processes such as oxidative phosphorylation, thermogenesis, the citrate cycle, cardiac muscle contraction, glycolysis/gluconeogenesis, retrograde endocannabinoid signaling, metabolism of pyruvate, amino sugar, nucleotide sugar, and starch and sucrose, which are closely associated with energy metabolism.

**Figure 9:**
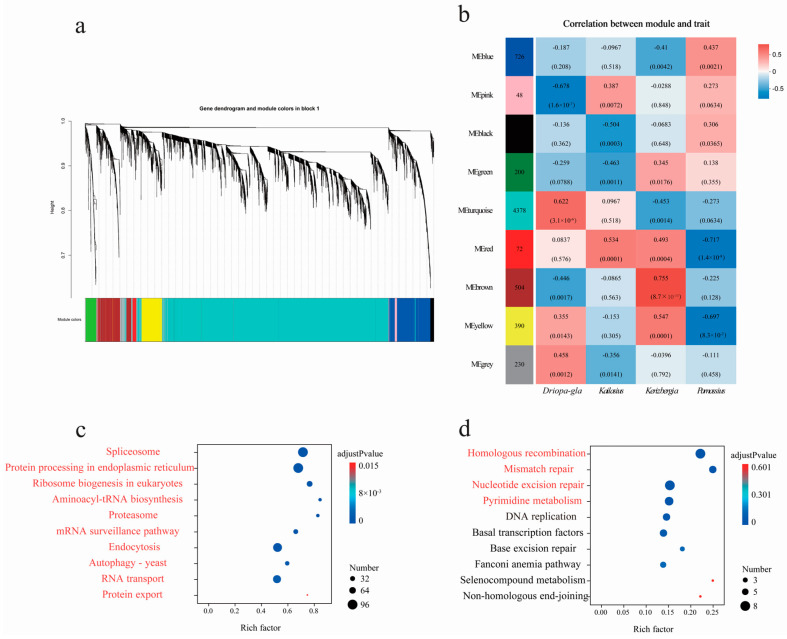
(**a**) Hierarchical clustering tree (gene dendrogram) showing nine modules of genes co-expressed by WGCNA analyses using *P. cephalus* as the reference genome. The major tree branches constitute nine modules, labeled with different colors. (**b**) Module–locality relationship (each row represents a module, each column represents a specific sampling locality, and the correlation coefficient between module and locality is represented by the value in each cell at the row–column intersection, with the *p*-value shown in parentheses. (**c**,**d**) KEGG enrichment analyses of the genes in the turquoise and brown modules, respectively. For each module, only the top 10 pathways with the most significant enrichment are shown.

The results revealed that the differentially expressed genes were clustered into nine major modules (Figure 9) using *P. cephalus* as the reference genome. Among them, two modules (turquoise and brown) were significantly associated with most subgenera with high Spearman correlation coefficients. Among them, two modules (turquoise and brown) contained large number of genes and were significantly associated with several subgenera with high Spearman correlation coefficients, both of which were also moderately correlated with the subgenus *Kailasius* at median altitudes. The turquoise module, comprising 4378 genes, was significantly positively correlated with *P. glacialis* (Subgenus *Driopa*) at low altitudes (Spearman correlation coefficient = 0.62, adjusted *p*-value = 3.06 × 10^−6^), and negatively correlated with the subgenera *Kerizberga* and *Parnassius* at relatively high altitudes (Spearman correlation coefficient ≤ −0.273, adjusted *p*-value ≤ 6.34 × 10^−2^). In contrast, the brown module, containing 504 genes, was positively correlated with the subgenus *Kerizberga* at relatively high altitudes (Spearman correlation coefficient = 0.755, adjusted *p*-value = 8.72 × 10^−10^), while negatively correlated with *P. glacialis* at low altitudes (Spearman correlation coefficient = −0.446, adjusted *p*-value = 1.68 × 10^−3^) (Figure 9). The KEGG enrichment analysis revealed that the genes in the turquoise module mainly participated in processes such as spliceosome, endoplasmic reticulum protein processing, ribosome biogenesis, proteasome, the mRNA surveillance pathway, RNA transport, and protein export, closely related to cell growth and development, and the genes in the brown module were primarily involved in functions such as homologous recombination, mismatch repair, nucleotide excision repair, and pyrimidine metabolism, closely related to genetic recombination and DNA repair (Table S7).

### 3.6. RNA-Seq Validation Using RT-qPCR

**Figure 10:**
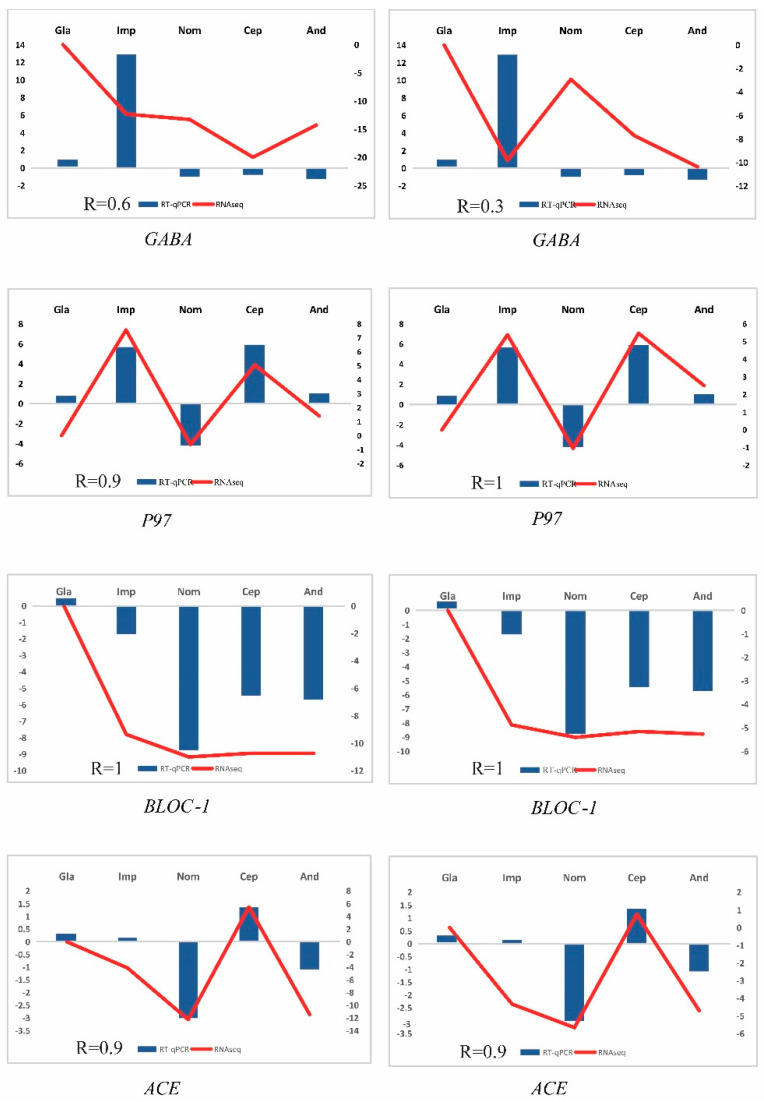
Validation expression patterns in *P. glacialis* and *P. cephalus* representative samples determined by qPCR. The left ordinate represents the qPCR-based expression levels, and the right ordinate represents the RNA-seq-based expression levels.

In order to validate the RNA-seq, a total of 10 genes (GABA receptor modulator (GABA), melanoma-associated antigen p97 (P97), V-type H^+^-transporting ATPase subunit F (ATPeV1F), mRNA-decapping enzyme subunit 2 (DCP2), glyoxylate/hydroxypyruvate reductase (GRHPR), ubiquitin-conjugating enzyme E2 (E2I), cyclin-dependent kinase 2 (CDK2), biogenesis of lysosome-related organelles complex 1 subunit 1 (BLOC-1), peptidyl-dipeptidase A (ACE), and myosin light chain 6 (MLC6)) were selected for testing using RT-qPCR. The results for the five representative samples confirmed the consistency of the gene expression pattern. The correlations between the RNA-seq and RT-qPCR data were extremely strong for eight genes (P97, BLOC-1, ACE, ATPeV1F, GRHPR, E2I, CDK2, and MLC6) (R > 0.80), while the correlations were less strong for the other two genes (GABA and DCP2) (with a range of R values from 0.20 to 0.60) (Figure 10). These discrepancies might be related to the methodological difference between RNA-seq and RT-qPCR, which seemed to be common in the transcript-level analyses. Nonetheless, the variation tendencies in the RNA-seq data curve and the RT-qPCR histogram were mostly similar, suggesting that our RNA-seq and RT-qPCR data analyses were reliable.

## 4. Discussion

Although research based on individual environmental factors has enhanced our understanding of the mechanisms by which crucial environmental factors influence the growth and development of insect groups [8,41], the methodology fails to fully capture the combined effects of multiple environmental factors in nature. It can only serve as a partial foundation for comprehending the adaptive mechanisms of insects under particular environmental factors. Moreover, increasing evidence has indicated that the intricate adaptation and evolution of insects in high-altitude environments can be attributed to the synergistic effect of numerous pathways and genes [42,43].

The genus *Parnassius*, one of the representative insect groups with high variation in altitudinal distribution, is an ideal model organism for deciphering the mechanisms of high-altitude adaptation [18,22]. Herein, multiple large-scale transcriptomic interpretation strategies were employed for the first time to decipher the interspecific and intraspecific transcriptomic expression patterns of *Parnassius* species at the whole genome-wide level. Considering the different phylogenetic distances of the different species derived from the reference genome, both the chromosome-level genomic data of *P. glacialis* and *P. cephalus* from our laboratory were firstly used as the references to alleviate the effect of different genetic backgrounds on the quantification of the gene expression matrix based on a single reference genome. Secondly, MRM were performed to assess the relative contributions of altitude, geographic location, and host plant on the gene expression patterns, and the results indicated that altitude change was shown to be the most significant factor in explaining the variation in gene expression. Then, large-scale transcriptomic analyses (including those of DEGs, KEGG enrichment, and WGCNA) were carried out between groups sampled at divergent altitudes (i.e., LA, MA, and HA), regardless of genetic backgrounds. Furthermore, featured modules from the WGCNA that contained large number of genes were significantly correlated to the subgenera (groups) of low and high altitudes while moderately correlated to those of median altitudes were focused on to decipher the potential adaptive mechanisms of different subgenera in the genus *Parnassius* to different altitudinal environments.

In the present study, though the variations in the number of DEGs and those of enriched pathways were found in each pairwise comparison or featured module, the results based on the analyses of the DEGs, KEGG enrichment, and WGCNA generally showed a series of shared pathways related to metabolism, genetic information processing, and cellular homeostasis. When compared to those of the low- and moderate-altitude groups, the up-regulated genes for the high-altitude *Parnassius* species or populations were primarily involved in the pathways of energy metabolism (i.e., oxidative phosphorylation, glycolysis/gluconeogenesis, the citric acid cycle, and the metabolism of various substances), which could facilitate the energy needs and maintain body temperature by promoting energy supply through digesting nutrients at low temperature, thereby enhancing the adaptation capability to the high-altitude environments [44,45]. Previous studies suggested that short-term hypobaric hypoxia induced an oxidative stress rather than an energy crisis in *L. migratoria* thoracic muscles, and that efficient utilization of aerobic metabolism could help Tibetan locusts conquer hypoxia [6,7]. The metabolism was also shown to be the most represented pathway (i.e., carbohydrate metabolism and lipid metabolism) in the Tibetan ghost moth *T. pui* exposed to short-term low-altitude conditions [8]. The findings above all indicated that insects could adapt to short-term hypoxic or normoxic pressure by modulating basic metabolic processes (i.e., energy metabolism); however, enhanced energy metabolism could reflect long-term adaptive evolution for high-altitude *Parnassius* species or populations, probably different from the short-term response to hypoxic or normoxic pressure. Moreover, several pathways associated with immune defense, genetic recombination and DNA repair, and hormone biosynthesis were also shown to be probably activated in the high-altitude *Parnassius* groups, likely facilitating anti-pathogen activity and anti-ultraviolet radiation and promoting reproduction for the *Parnassius* species at high altitudes, respectively.

Interestingly, other functional pathways (i.e., protein processing in the endoplasmic reticulum, mRNA surveillance pathway, spliceosome, endocytosis, and proteasome), related to cellular homeostasis, erroneous protein degradation, and cell apoptosis, were also significantly activated in either the high-altitude or low-altitude *Parnassius* butterflies. These findings indicated that all *Parnassius* species herein had adapted locally to disparate ecological zones at different altitudes through long-term adaptive evolution. Similar cases were also found in previous studies on other arthropods, such as the migratory locust (*L. migratoria*), the ghost moth (*T. pui*), and three-year-old crabs (*Eriocheir sinensis*) [6,7,8,46].

In contrast, the pathways related to cell growth and developmental regulation were consistently inhibited for the higher-altitude butterflies, likely due to the low level of oxygen and temperature at high altitudes. Such physiological processes are beneficial to conserve energy for maintaining normal cell activities, ensuring survival and reproduction [47,48]. This case indicates that high-altitude *Parnassius* butterflies undergo a highly intricate systemic process in gene expression regulation to enhance their adaptability to the plateau’s extreme environment. Similar scenarios have been observed in other insect and animal groups subjected to hypoxia or low-temperature treatments [2,6,42].

In summary, this study attempted to conduct, for the first time, a comparative transcriptomic analysis of *Parnassius* butterflies with reference genomes, including differentially expressed genes (DEGs), KEGG enrichment pathways, WGCNA, and others, at the interspecific and intraspecific levels. The results indicated that distributional altitude difference was the primary factor influencing the variation in gene expression patterns, although the effects of genetic background, gender, developmental stage, and larval host plants should still be considered. Additionally, the comparative transcriptomic analysis suggested that the *Parnassius* butterflies were activated in metabolic pathways related to energy metabolism, DNA repair, and immune defense as their distributional altitudes increased, and the cases were strongly shown by a close correlation between these pathways and the altitudes of the butterfly habitats.

## Data Availability

Both the genome and transcriptome sequencing data of *P. glacialis* were deposited into GenBank with the BioProject numbers PRJNA893814, PRJNA916644, PRJNA1125653, and the *P. cephalus* genome file has been uploaded to the figshare website (10.6084/m9.figshare.26058301).

## Supplementary Materials

The following supporting information can be downloaded at: https://www.mdpi.com/article/10.3390/genes15081013/s1, Table S1: Sampling materials and relevant information. Table S2: Primers used for quantitative real-time PCR. Table S3: Statistics of transcriptome sequencing raw data. Table S4: Statistics of MRM in this study. Table S5: The top 10 statistics of KEGG pathways in interspecific comparisons. Table S6: The top 10 statistics of KEGG pathways in intraspecific comparisons. Table S7: KEGG enrichment pathways of representative modules. Table S8. The number of DEGs in each pairwise comparison for interspecific analysis (|log_2_FoldChange| > 2). Table S9. The number of DEGs in each pairwise comparison for intraspecific analysis (|log_2_FoldChange| > 2). Figure S1: The number of differentially expressed genes for each pairwise comparison.
