# Supplementary material for: Interspecific and Intraspecific Transcriptomic Variations Unveil the Potential High-Altitude Adaptation Mechanisms of the Parnassius Butterfly Species
Source: Genes (Basel). 2024 Aug 1;15(8):1013. doi: 10.3390/genes15081013 (PMC11354221; doi:10.3390/genes15081013)
Supplement: Supplementary file 1 [file genes-15-01013-s001.zip › Supplementary_Figure.pdf]

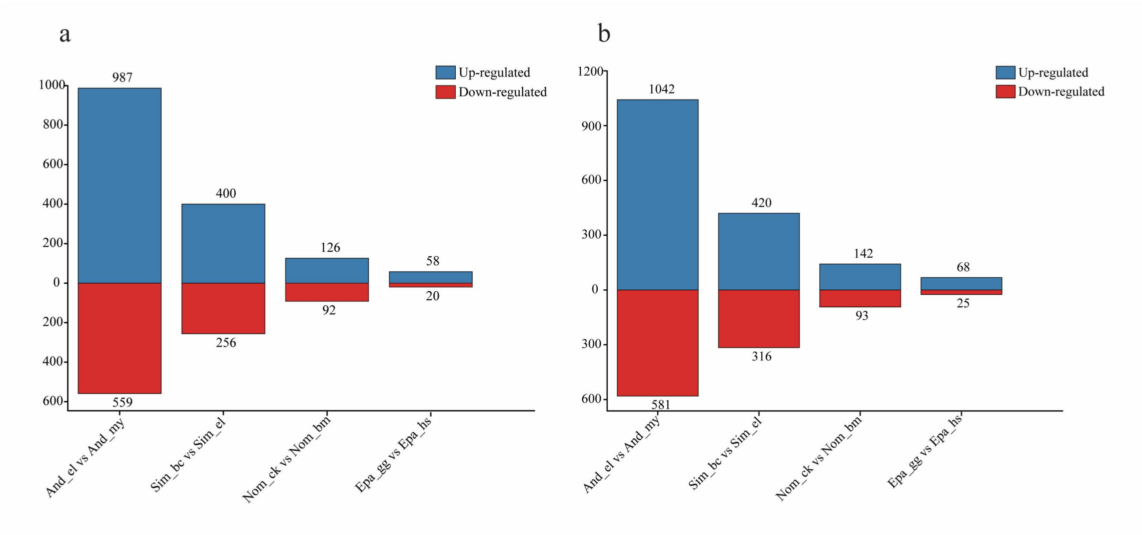

Figure S1. The number of differentially expressed genes for each pairwise comparison. Bar chart diagrams showing overlaps of DEGs with increased (red) or decreased (blue) transcript abundance in three pairs of comparisons (a: *P. glacialis* is used as the reference genome; b: *P. cephalus* is used as the reference genome).
